# Attention deficits revealed by passive auditory change detection for pure tones and lexical tones in ADHD children

**DOI:** 10.3389/fnhum.2015.00470

**Published:** 2015-08-26

**Authors:** Ming-Tao Yang, Chun-Hsien Hsu, Pei-Wen Yeh, Wang-Tso Lee, Jao-Shwann Liang, Wen-Mei Fu, Chia-Ying Lee

**Affiliations:** ^1^Department of Pediatrics, Far Eastern Memorial HospitalNew Taipei City, Taiwan; ^2^Institute of Pharmacology, National Taiwan UniversityTaipei, Taiwan; ^3^Brain and Language Laboratory, Institute of Linguistics, Academia SinicaTaipei, Taiwan; ^4^Department of Psychology, Lancaster UniversityUK; ^5^Department of Pediatrics, National Taiwan University HospitalTaipei, Taiwan; ^6^Laboratory for Cognitive Neuropsychology, National Yang-Ming UniversityTaipei, Taiwan; ^7^Institute of Cognitive Neuroscience, National Central UniversityJhongli, Taiwan

**Keywords:** attention deficit-hyperactivity disorder, event-related potential, passive auditory discrimination, mismatch negativity, P3a, late discriminative negativity

## Abstract

Inattention (IA) has been a major problem in children with attention deficit/hyperactivity disorder (ADHD), accounting for their behavioral and cognitive dysfunctions. However, there are at least three processing steps underlying attentional control for auditory change detection, namely pre-attentive change detection, involuntary attention orienting, and attention reorienting for further evaluation. This study aimed to examine whether children with ADHD would show deficits in any of these subcomponents by using mismatch negativity (MMN), P3a, and late discriminative negativity (LDN) as event-related potential (ERP) markers, under the passive auditory oddball paradigm. Two types of stimuli—pure tones and Mandarin lexical tones—were used to examine if the deficits were general across linguistic and non-linguistic domains. Participants included 15 native Mandarin-speaking children with ADHD and 16 age-matched controls (across groups, age ranged between 6 and 15 years). Two passive auditory oddball paradigms (lexical tones and pure tones) were applied. The pure tone oddball paradigm included a standard stimulus (1000 Hz, 80%) and two deviant stimuli (1015 and 1090 Hz, 10% each). The Mandarin lexical tone oddball paradigm’s standard stimulus was /yi3/ (80%) and two deviant stimuli were /yi1/ and /yi2/ (10% each). The results showed no MMN difference, but did show attenuated P3a and enhanced LDN to the large deviants for both pure and lexical tone changes in the ADHD group. Correlation analysis showed that children with higher ADHD tendency, as indexed by parents’ and teachers’ ratings on ADHD symptoms, showed less positive P3a amplitudes when responding to large lexical tone deviants. Thus, children with ADHD showed impaired auditory change detection for both pure tones and lexical tones in both involuntary attention switching, and attention reorienting for further evaluation. These ERP markers may therefore be used for the evaluation of anti-ADHD drugs that aim to alleviate these dysfunctions.

## Introduction

Attention-deficit/hyperactivity disorder (ADHD), a neuro developmental disorder, is characterized by symptoms of inattention (IA) and/or hyperactivity-impulsivity apparent and beginning before the age of 12 (American Psychiatric Association, 2013). The two symptom domains of ADHD are inattentiveness and hyperactivity/impulsivity (HI). The prevalence of ADHD in primary school children was 11.4% (Willcutt, 2012). Attention is a core ability for our daily life. Individuals, especially children, with attention deficits may also experience academic and social problems. Indeed, the symptom of IA in children with ADHD has been correlated with all domains of school dysfunction (Wu and Gau, 2013). To be more specific, Abdo et al. (2010) found that children with ADHD showed lower performance in the Speech in Noise test, Dichotic listening test, and Frequency Pattern test, when compared to children with dyslexia and age-matched controls. These findings suggest a relationship between poorer performance on auditory processing and IA. Similarly, Bellani et al. (2011) found that children with ADHD suffer from language disturbances, including speech discrimination, listening comprehension, verbal and spatial working memory, discourse analysis, pragmatic aspects of communication, and language comprehension. Thus, the early identification of children with ADHD, thereby allowing early intervention, is an important issue for the prevention of school dysfunction in late childhood and adolescence.

Currently, the diagnosis and classification of ADHD primarily depend on the clinical observation of patients’ behavior and on diagnostic interviews, based on the Diagnostic and Statistical Manual of Mental Disorders (DSM)-IV criteria before 2013 and DSM-five criteria since 2013 (American Psychiatric Association, 2013). In Taiwan, the Chinese version and the original English version of the Swanson, Nolan, and Pelham rating scale version IV (SNAP-IV) have been shown to be reliable and valid instruments for rating ADHD-related symptoms (Gau et al., 2008, 2009). Neuropsychological tasks, such as the attention network test (Fan et al., 2002) and the continuous performance test (CPT; Conners and Staff, 2004), which measures reaction time (RT) and accuracy, have been widely used to evaluate attentional control and processing efficiency in patients with ADHD.

However, behavioral measurements of auditory and visual attention often require the subject to provide an overt response and be motivated to cooperate, conditions that are often unmet in children, especially those with ADHD. In recent years, a growing body of research has utilized the event-related potential (ERP) as an alternative method, to evaluate attentional processing more directly. A set of ERP components, such as N1, P2, mismatch negativity (MMN), P3a and late discriminative negativity (LDN, or late MMN) or reorienting negativity (RON), have been identified as reflecting various stages of sensory and attentional processing (Barry et al., 2003; Escera and Corral, 2007; Jakoby et al., 2011; Näätänen et al., 2012). For example, MMN is an ERP index for automatic auditory change detection. It is typically obtained in a passive auditory oddball paradigm, in which infrequent auditory changes (deviant stimuli) were embedded in a chain of repetitive homogenous sounds (standard stimuli). In adults, MMN is observed as a frontal-distributed negativity peaking between 100 and 250 ms after stimulus onset by subtracting the ERPs for the standard stimulus from those for the deviant stimuli (Näätänen et al., 2007). MMN amplitude increases and peak latency decreases, as the discriminability of the standard and deviant sounds rises (Näätänen et al., 2007). Thus, MMN has become a valuable neural marker for evaluating auditory discrimination ability not only in adults but also in children and infants, given that overt attention is not required for its elicitation.

However, although MMN is well established in adults, studies often report a positive mismatch response (P-MMR), instead of MMN, in infants and children (Maurer et al., 2003; Lee et al., 2012; Cheng et al., 2013). Studies demonstrating the developmental change from P-MMR to adult-like MMN with age suggest that P-MMR might reflect an immature brain response (He et al., 2007; Ahmmed et al., 2008; Lee et al., 2012). Maurer et al. (2003) noted that young children’s P-MMR increased with the degree of phoneme deviance. Some studies thus suggest that the P-MMR might be an analog of P3a in adults—that is, a response to reflect involuntary attention switching to a task-irrelevant novel sound (Kushnerenko et al., 2002; Shestakova et al., 2003; He et al., 2009). In adult data, MMN is often followed by P3a, which usually peaks between 300–400 ms maximally over the frontal-central scalp. The amplitude of P3a increases in magnitude as a function of stimulus change (Picton, 1992). In general, P3a elicitation depends on whether the deviant stimulus is sufficiently different (as indexed by the preceding MMN) to capture attention, albeit at an unconscious level (Jakoby et al., 2011). As a result, P3a is believed to reflect an involuntary switch of attention upon adequate change detection (Escera et al., 1998, 2000). Thus, the presence of P-MMR in infants or young children might be due to the absence of MMN overlapping with P3a.

In addition to MMN and P3a, LDN, which can also be elicited by deviant stimuli in the passive oddball paradigm in late time window, has different characteristics from the classic MMN. LDN, usually occurring between 400–700 ms after change onset with a fronto-central distribution, is often found in young children, and its amplitude tends to decrease with age (Cheour et al., 2001; Korpilahti et al., 2001). The functional significance of LDN is still far from clear. Bishop et al. (2010) found that LDN was larger for small deviants than for large deviants, and suggested on this basis that the LDN reflects the further processing of auditory stimuli when the salient features of the stimulus are difficult to discriminate. Neuhoff et al. (2012) suggested that further auditory processing, indexed by LDN, might be associated with higher cognitive processes, such as attention-related processes, letter-speech sound integration, and long-term memory. Mueller et al. (2008) showed that LDN was mainly found in the attended condition and was considerably reduced in children and absent in adults under the unattended condition. They suggested on this basis that LDN is affected by active stimulus detection and related to the counting of deviant stimuli (Mueller et al., 2008). Näätänen et al. (1982) speculated that LDN might be associated with “sensitization processes,” so that a repeated stimulus might be noticed subliminally. Therefore, LDN may be regarded as a form of automatic preparation to detect additional stimuli (Näätänen et al., 1982; Mueller et al., 2008). Others have suggested that LDN may reflect the attention reorienting back to the original task, similar to RON, which reflects attentional allocation after distraction and general reorientation of attention (Schröger and Wolff, 1998; Escera et al., 2000; Munka and Berti, 2006).

To summarize, at least three ERP components—MMN (or P-MMR), P3a, and LDN—could be identified to index various stages of attentional processes during the passive auditory discrimination task, including pre-attentive change detection, involuntary attention switching, and attention reorienting. Some MMN studies have suggested that children with ADHD exhibit an auditory discrimination deficit (Kemner et al., 1996; Winsberg et al., 1997; Kilpeläinen et al., 1999b); however, this assertion remains controversial. For example, Kemner et al. (1996) found no MMN, but a P3a difference between children with ADHD and age-matched controls. Kilpeläinen et al. (1999b) identified both MMN and LDN in 9-year-old children with or without ADHD; only LDN was significantly reduced in the ADHD group. They also found a strong frontal lobe contribution to the generation of LDN and suggested on that basis that ADHD may involve deficits in frontally mediated auditory sensory memory. Winsberg et al. (1993) found decreased amplitude and increased latency of MMN in children with ADHD along with a trend toward normalization by methylphenidate; however, their later study found no difference in MMN between children with ADHD and controls, nor was MMN found to be affected by methylphenidate (Winsberg et al., 1997). Oades et al. (1996) showed reduced MMN latency in children with ADHD and suggested that these children processed perceptual information faster than normal controls. Huttunen-Scott et al. (2008) found no MMN difference, but larger P3a, in an ADHD group than in controls. They argued that P3a, as an indication of attention switch, would be expected in children with ADHD, because of their known distractibility.

However, it remains controversial whether children with ADHD will reveal deficits in pre-attentive change detection (indexed by MMN), involuntary attentional switch (indexed by P3a), or attention reorienting for further auditory processing (indexed by LDN) during passive auditory discrimination. More important, most previous studies applied non-speech stimuli, rather than speech sounds. This study thus aimed to look for the neural markers of attentional deficit in children with ADHD using MMN, P3a, and LDN, which can be elicited in the passive auditory oddball paradigm from early to late time windows, as potential indicators of auditory change detection with both lexical and pure tones as stimuli. The size of deviance in both Mandarin lexical tones and pure tones will be manipulated. Modulation of different responses to speech and non-speech stimuli by attention has been noted (Shtyrov et al., 2012); therefore, responses to lexical and pure tones may not be the same. Mandarin is a tonal language with four lexical tones: a high-level tone (T1), a high-rising tone (T2), a low-dipping tone (T3), and a high-falling tone (T4). Among these, T2 is acoustically more similar to T3 than to T1 in terms of pitch contour and direction. Previous behavioral studies using tonal discrimination and identification data have suggested that the T2 and T3 pair are acoustically similar and more often confused with each other, compared to other tonal pairs (Wong et al., 2005; Tsao, 2008). Correspondingly, previous studies have demonstrated that the T1/T3 pair elicited greater and earlier MMN than the T2/T3 pair did (Chandrasekaran et al., 2007; Cheng et al., 2013). A recent magnetoencephalography (MEG) study for Mandarin lexical tonal changes demonstrated that mismatch fields were generated in bilateral superior temporal gyri and activated a set of frontal generators that were thought to be associated with various top-down mechanisms for attentional modulation (Hsu et al., 2014). However, it remains to be investigated whether ADHD will reveal different effects on MMN, P3a, and LDN in both linguistic (lexical tone) and non-linguistic (pure tone) changes.

## Materials and Methods

### Participants and ADHD Case Identification

Fifteen native Mandarin-speaking children with ADHD and sixteen age-matched controls were enrolled in this study (Table 1). Their ages ranged between 6 and 15 years.

**Table 1:** **Demographic characteristics, ADHD symptom scores, and continuous performance test between the control and ADHD groups**.

		Control (*n* = 16)	ADHD (*n* = 15)	*p* value
	Age	10.84 ± 2.87	9.15 ± 2.00	0.069
	Gender (M/F)	6/10	12/3	0.017^#^
DSM	IA (Parent)	1.83 ± 1.40	7.47 ± 1.30	<0.001***
	IA (Teacher)	1.42 ± 1.38	7.20 ± 2.01	<0.001***
	HI (Parent)	0.25 ± 0.45	4.73 ± 2.94	<0.001***
	HI (Teacher)	0.67 ± 1.50	5.00 ± 2.56	<0.001***
SNAP	IA (Parent)	7.92 ± 4.40	20.00 ± 3.32	<0.001***
	IA (Teacher)	7.00 ± 3.36	19.87 ± 4.49	<0.001***
	HI (Parent)	3.67 ± 2.87	12.93 ± 5.66	<0.001***
	HI (Teacher)	2.92 ± 3.94	14.53 ± 7.75	<0.001***
CPT	Confidence index	35.61 ± 14.18	44.63 ± 13.74	0.083^m^
	Omission	45.87 ± 5.88	47.48 ± 6.30	0.468
	Commission	35.29 ± 10.10	43.45 ± 10.18	0.033*
	Hit RT	53.72 ± 10.22	54.42 ± 7.91	0.832
	Hit RT SE	44.04 ± 7.96	48.86 ± 7.18	0.088^m^
	Variability	42.74 ± 7.94	48.56 ± 7.77	0.049*
	Detectability (d’)	39.62 ± 11.70	44.97 ± 8.57	0.160
	Response style (B)	50.67 ± 11.09	48.68 ± 7.64	0.569
	Perseverations	45.16 ± 2.68	46.47 ± 7.13	0.498
	Hit SE block change	46.80 ± 6.16	48.97 ± 6.91	0.364
	Hit RT ISI change	43.02 ± 6.06	49.29 ± 6.81	0.011*
	Hit SE ISI change	46.32 ± 5.11	50.03 ± 8.60	0.152

Gender (M/F) is presented as the number of participants. Confidence index is expressed as chances out of 100. All CPT measures are presented as T-score of the general population. All other data are presented as mean ± standard deviation. *ADHD, attention deficit hyperactivity disorder; CPT, continuous performance test; DSM, Diagnostic and statistical manual of mental disorders; HI, Hyperactivity/Impulsivity subscale; IA, Inattention subscale; ISI, inter-stimulus interval; RT, reaction time; SE, standard error; SNAP, Swanson, Nolan, and Pelham, version IV Rating Scale*. #*p* < 0.05, compared with control group using Pearson Chi-Square test. ^m^*p* < 0.1; *p < 0.05; ***p < 0.001, compared with control group using Student’s *t*-test.

All participants with ADHD were recruited from the outpatient clinics at the Far Eastern Memorial Hospital, New Taipei City, Taiwan. They met the DSM-5 criteria for the diagnosis of ADHD (American Psychiatric Association, 2013), and none had taken any ADHD drug before participating in the experiment (i.e., they were ADHD medication-naïve). Of the children with ADHD, 7 (46.7%) children met the criteria for the combined subtype of ADHD, 7 (46.7%) for the inattentive subtype, and 1 (6.7%) for the hyperactive/impulsive subtype; additionally, 5 (33.3%) of the 15 children with ADHD also met the criteria for oppositional defiant disorder. The control children were age-matched to the ADHD group and were recruited from ordinary primary schools and outpatient clinics.

Electroencephalography (EEG) and audiometric testing were required for every participant to exclude epilepsy and hearing impairment, respectively. All the children had normal or corrected-to-normal vision. No participant had any psychiatric or neurological disorders, and none was taking any medication. In Taiwan, the Committee Responsible for Identification and Placement of Gifted and Disabled Students, which consists of teachers, clinical psychologists and medical doctors, is responsible for the identification and placement of children with learning and reading disabilities to ensure that those children can receive special education in school. None of the children participating in the present study had been identified as learning or reading disabled.

Both ADHD and normal control children were evaluated for ADHD-related symptoms using the Chinese version of SNAP-IV (Gau et al., 2008, 2009) and computerized CPT (Conners and Staff, 2004).

SNAP-IV, a 26-item questionnaire using a four-point Likert-type scale, to be completed by each participant’s parents and teachers, has been shown to be a valid instrument for rating ADHD-related symptoms (Gau et al., 2008); it consists of IA, HI, and Oppositional subscales. The 26 items include 18 for ADHD symptoms (9 for the IA subscale and 9 for the HI subscale) and 8 for oppositional defiant disorder symptoms as defined in the DSM-5. Each item is scored on a 0–3 scale (0 = not at all, 1 = just a little, 2 = quite a bit, and 3 = very much). A score of 2 (quite a bit) or 3 (very much) was defined as indicating the symptomatic presence of this behavior; otherwise, the symptom in question was considered absent according to DSM diagnostic criteria.

The CPT, developed by Beck et al. (1956), is among the most-widely-used neuropsychological tests for patients with ADHD. The computerized CPT (Conners’ CPT II, Multi-Health Systems, Inc., North Tonawanda, NY, USA) runs for 14 min and evaluates attention and impulsivity (Conners and Staff, 2004). Participants pressed a space bar when any letter except “X” appeared on a computer screen. Multiple dependent measures were gained, including omissions, commissions, hit RT, variability of standard error (SE), and detectability. The confidence index gathering all dependent measures gives a chance out of 100 that a clinical ADHD problem exists. The raw values of all measures except the confidence index are converted to T-scores and percentiles by comparing the respondents to those in the normal group of the same gender and the same age group. T-scores were gathered for analysis.

IA and HI subscale scores for both DSM and SNAP from both parents and teachers were significantly higher in the ADHD group (Table 1). CPT showed significantly higher commission error, variability, and hit RT inter-stimulus interval (ISI) change, and marginally higher confidence index and hit RT standard error in the ADHD group. The higher ADHD symptom scores and abnormal CPT measures corroborated the essential features expected for an ADHD group.

The study was approved by the Research Ethics Review Committee of Far Eastern Memorial Hospital. Informed consent forms were obtained from all participants and their parents.

### Stimuli

Two sets of stimuli were designed and used in two separate experiment sessions, one for lexical tone changes and the other for pure tone changes. For the lexical tone condition, we adopted the same stimuli and experimental procedure as in previous studies by us (Lee et al., 2012; Cheng et al., 2013). For the lexical tone condition, three Mandarin syllables, /yi1/ (T1: high-level, F0 around 230 Hz), /yi2/ (T2: high-rising, F0 from 180 to 200 Hz) and /yi3/ (T3: low-dipping, F0 descends from 100 to 80 Hz and then rises to 100 Hz again), all sharing the same vowel, /i/, but with different tonal contours, were applied. These three syllables are all meaningful in Mandarin Chinese. We assigned T3 as the common standard, and T1 and T2 as large and small deviants, respectively. A female native Mandarin speaker produced all lexical tone stimuli, which were recorded at 16 bits, with a 44 kHz sampling rate. For the pure tone condition, we assigned a 1000 Hz pure tone as the common standard, and 1015 and 1090 Hz pure tones as small and large deviants, respectively. The intensity and duration of all the stimuli were normalized to 70 dB and 250 ms, respectively, using Sony Sound Forge 9.0 software.

### Procedure

Participants were seated in a soundproof and electrically shielded room. They were instructed to play a computer puzzle game called “super-box” silently while listening passively to auditory stimuli. The stimuli were presented at 70 dB through two loudspeakers located approximately 75 cm in front of the participant. Changes in pure tone and in lexical tone were presented in two separate experimental sessions for each participant. Each experimental session consisted of an initial 20 standard trials and 1000 following trials, 20% deviant (10% large deviant and 10% small deviant) and 80% standard. All stimuli were pseudo-randomized with at least two successive standards between deviants. “To fully control for sequence effect, only those standard trials that were followed by at least two standards were analyzed” (Lee et al., 2012). Each stimulus (each trial) lasted for 250 ms, with an inter-trial interval of 500 ms. Participants were allowed to take a break if needed. Each experiment ran for about 30 min.

### Data Recording, Preprocessing and Analysis of Event-Related Potentials

As described in our previous study (Lee et al., 2012), EEG data were recorded by QuickCap (Neuromedical Supplies, Sterling, CA, USA), which consists of 32 Ag/AgCl electrodes referenced online to the average of the left and right mastoids with impedance below 10 KΩ. Electrooculography data were recorded through electrodes placed above and below the left eye (vertical) and at the outer canthi (horizontal). The EEG signal amplified with SynAmps2 (Neuroscan, Inc., Charlotte, NC, USA) was continuously recorded and digitized at a rate of 500 Hz, with the band-pass set at 0.1–100 Hz. For offline analysis, the continuous EEG data were divided into epochs of 800 ms in length, starting 100 ms before the stimulus onset. The pre-stimulus interval (−100 to 0 ms) was used for baseline correction. Before artifact rejection, the data were band-pass filtered to 1–30 Hz (zero-phase shift mode, 12 dB). Ocular artifact rejection excluded trials with voltage variations larger than ±100 μV in electrooculography; trials with voltage variations larger than ±100 μV in at least one of the channels were also rejected. In addition, we excluded from the averages the standard trials that immediately followed the deviant trials. For lexical tone, the mean numbers of trials for standard, large deviant, and small deviant were 533.3, 88.7, and 89.4, respectively, for the ADHD group and 547.3, 91.4, and 91.8, respectively, for the control group. For pure tone, the mean numbers of trials for standard, large deviant, and small deviant were 544.3, 93.9, and 92.8, respectively, for the ADHD group and 564.6, 96.7, and 94.9, respectively, for the control group. There was no statistically significant difference between groups in the trials (all *p* > 0.1).

### Statistical Analysis

#### Behavioral Data

For the behavioral and neuropsychological tests, the Student’s *t*-test was used to compare the ADHD symptom scores (DSM, SNAP) and CPT scores between the control group and the ADHD group. All data were analyzed using SPSS Statistics, version 19.0 (IBM Inc., Somers, NY, USA). All tests were two-tailed; *p* < 0.05 were considered statistically significant.

#### ERP Data Analysis

##### Cluster-based analyses

To evaluate the spatiotemporal differences between the standards and the deviants, a cluster-based random permutation analysis (Maris and Oostenveld, 2007) was conducted in mean amplitudes of 12 successive epochs of 50 ms each, from 100 to 700 ms. A moving time window analysis like this one has been used in developmental studies (Lee et al., 2012; Cheng et al., 2013) to address the fact that MMR patterns may change across age groups, types of stimulus, and sizes of deviance. Applying both the moving time window analysis and the cluster-based random permutation analysis allows us to identify when and where the standard and the deviant differ from each other for each group, and to effectively handle the multiple-comparisons problem. First, a simple dependent-samples *t*-test was performed for each electrode. All electrodes that exceeded a set significance level (alpha = 0.05) were identified and grouped into clusters. Any cluster that had fewer adjacent channels than three was excluded from subsequent analyses. For each cluster, a cluster-level test statistic was calculated by taking the sum of all the individual *t*-statistics within that cluster. Next, a null distribution was created by computing 1000 randomized cluster-level test statistics. Finally, the actually observed cluster-level test statistics were compared against the null distribution. If the summed *t*-value of the observed cluster fell into the highest or lowest 2.5th percentile (less than an alpha-level of 0.05, two-tailed), then the cluster was considered to be significant.

##### Conventional analyses for MMN, P3a, and LDN

**Figure 1:**
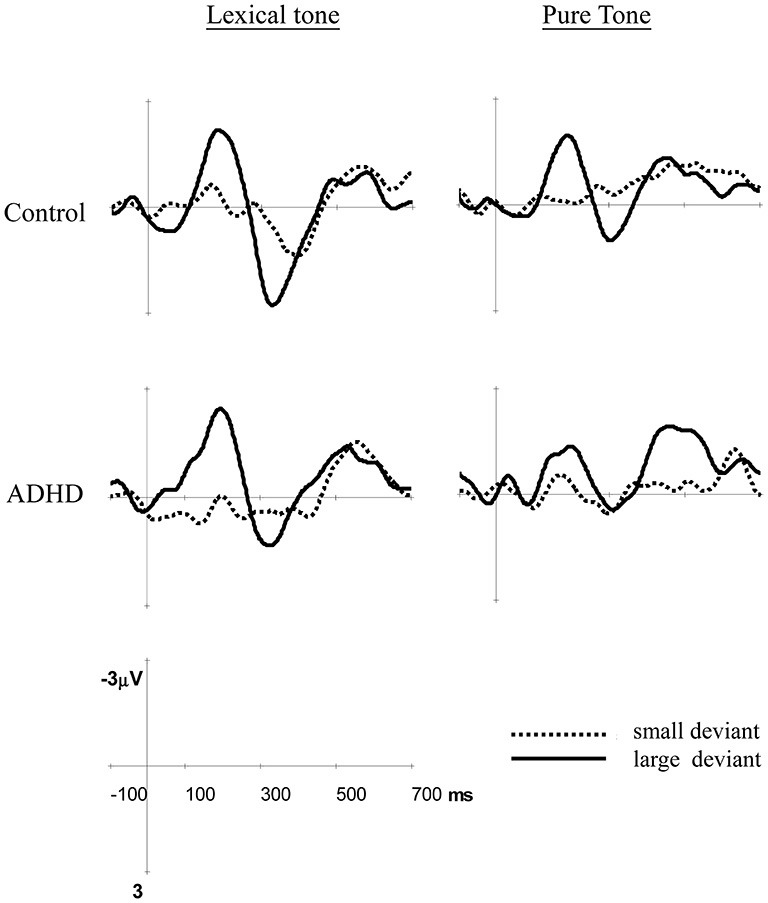
**Differential event-related potentials (ERPs) for lexical tones and pure tones in these two groups at Fz**. Small deviant: T2/T3 and 1015/1000 Hz; large deviant: T1/T3 and 1090/1000 Hz.

We also performed conventional single time-window analysis to reconfirm the results of the cluster-based analysis. Based on the visual inspection of difference waves in Figure 1 and the results of the cluster-based permutation analysis, mean amplitudes were measured for MMN (150–250 ms), P3a (300–400 ms), and LDN (450–650 ms) for lexical tone, and MMN (150–250 ms), P3a (250–350 ms), and LDN (350–650 ms) for pure tone. Mean amplitudes from these time windows were submitted to separate four-way analyses of variance (ANOVAs), with group (control and ADHD) as a between-subject factor, and condition (standard, large deviant, and small deviant), and electrodes (25 channels to cover the whole head: F3, F1, FZ, F2, F4, FC3, FC1, FCZ, FC2, FC4, C3, C1, CZ, C2, C4, CP3, CP1, CPZ, CP2, CP4, P3, P1, PZ, P2, and P4) as within-subject factors. Regardless of any significant main effect of group or interaction related to group, planned multiple comparisons were performed to examine whether the deviants departed significantly from standard in each group at each site, for both lexical tone and pure tone. In addition, we also examined whether the two groups showed significant differences in MMN, P3a, and LDN under large and small deviants at each site. For all ANOVAs, the Greenhouse-Geisser correction was applied wherever necessary to correct for violations of sphericity (Greenhouse and Geisser, 1959). Corrected *p*-values and original degrees of freedom were reported. While performing planned multiple comparisons, the Dunn-Šidák method was used to control Type I errors.

## Results

### Behavioral Measures

Children with ADHD showed significantly higher ADHD symptom scores (DSM, SNAP) when compared to the control group. Children with ADHD also showed higher CPT scores for commission, variability, and Hit RT ISI than the control group did (see Table 1).

### ERP Data

**Figure 2:**
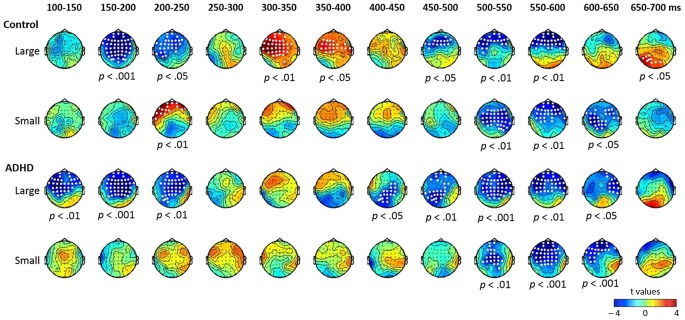
**Topographic maps of the differential ERPs for lexical tone in 12 successive time windows from 100 to 700 ms in control and ADHD groups**. The white dots in the topographies indicate the electrodes that showed a significant difference between the standard and deviant in the time window.

**Figure 3:**
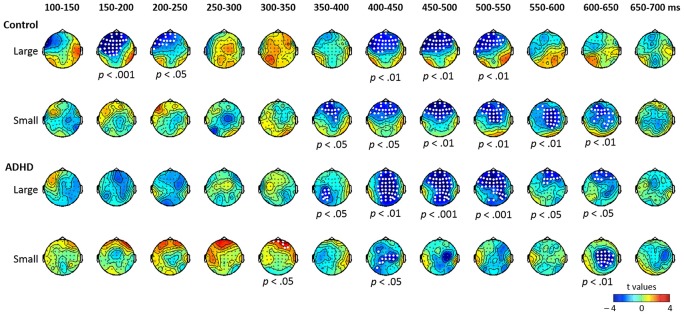
**Topographic maps of the differential ERPs for pure tone in 12 successive time windows from 100 to 700 ms in control and ADHD groups**. The white dots in the topographies represent the electrodes that showed a significant difference between the standard and deviant in the time window.

Figure 1 shows the mismatch responses at Fz by subtracting the standard from the two deviants in the control and ADHD groups for lexical and pure tones. The visual inspection of the differential waves revealed three ERP components, MMN (100–250 ms), P3a (250–400 ms), and LDN (400–700 ms), especially for the large deviant conditions (T1/T3 and 1090/1000 Hz) for both control and ADHD groups. Figures 2, 3 show topographic maps of the mismatch responses for each group to each deviant in 12 successive time windows from 100 to 700 ms for lexical tone and pure tone, respectively.

#### The Cluster-Based Permutation Analysis

##### Lexical tone contrasts in the control and ADHD groups

The results of the cluster-based permutation analysis for lexical tone are shown in Figure 2, which reveals several significant clusters for large and small deviants in both control and ADHD groups.

In the control group, the contrast between large deviant T1 and standard T3 yielded significant clusters in eight time windows (*p*s < 0.05; see the first row of Figure 2). There were significant negative clusters from 150 to 250 ms in the frontal-to-central electrodes, which may be considered to indicate MMN; significant positive clusters from 300 to 400 ms in frontal-to-central electrodes, which may be considered to indicate P3a; negative clusters from 450 to 600 ms in frontal-to-central electrodes, which may be considered to indicate LDN; and a significant positive cluster in posterior sites from 650 to 700 ms. The contrast between small deviant T2 and standard T3 yielded significant clusters in four time windows (*p*s < 0.05; see the second row of Figure 2), including a positive cluster between 200–250 ms in left frontal electrodes, which may be considered to indicate P-MMR, and negative clusters from 500 to 650 ms with frontal-to-central distribution.

In the ADHD group, the contrast between large deviant T1 and standard T3 yielded significant clusters in eight time windows (*p*s < 0.05), including negative clusters from 100 to 250 ms and from 400 to 650 ms. The contrast between small deviant T2 and standard T3 yielded three negative clusters from 500 to 650 ms (*p*s < 0.05).

##### Pure tone contrasts in the control and ADHD groups

The results of the cluster-based permutation analysis for pure tone are shown in Figure 3. In the control group, the contrast between large deviant and standard yielded significant clusters in five time windows (*p*s < 0.05): negative clusters from 150 to 250 ms and from 400 to 550 ms in-frontal-to-central sites, which may be considered to indicate MMN and LDN, respectively. Regarding the contrast between small deviant and standard, six time windows (from 350 to 650 ms; *p*s < 0.05) showed significant negative clusters with frontal-to-central distribution.

In the ADHD group, the contrast between large deviant and standard yielded negative clusters in six time windows (from 350 to 650 ms; *p*s < 0.05). Regarding the small deviant, three time windows showed significant clusters (*p*s < 0.05)—a positive cluster in the right frontal sites from 300 to 350 ms, and two negative clusters from 400 to 450 ms and from 600 to 650 ms, respectively, with central-to-posterior distribution.

To summarize the results of the cluster-based analysis, for the large lexical tone change, both control and ADHD groups showed statistically reliable MMN and LDN, yet only the control group showed a P3a. For the small lexical tone change, the control group revealed a P-MMR and a LDN, while the ADHD group showed only an LDN. As for pure tone, the large deviant elicited an MMN in the control group, but not in the ADHD. In the pure tone condition, LDNs were found in both groups, across large and small pure tone deviants.

##### Group effect for each contrast

**Figure 4:**
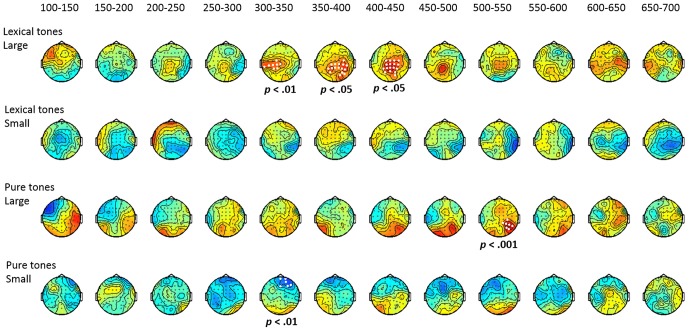
**Topographic maps of the group effect (control minus ADHD), based on cluster-based permutation analysis**.

To further evaluate the group effect, a cluster-based random permutation analysis was applied to examine if the mismatch responses of the ADHD group were significantly different from that of the control group (shown in Figure 4). The results revealed the presence of significant positive clusters from 300 to 450 ms (*p*s < 0.05) for large deviants of lexical tone contrasts, indicating that the control group elicited more positive P3a than the ADHD group did. For the small deviants of lexical tone contrasts, there was no significant difference between groups. Regarding the pure tone contrasts, the control group yielded a significant positive cluster from 500 to 550 ms (*p* < 0.001) in response to the large deviant, and a significant negative cluster from 300 to 350 ms (*p* < 0.01) in response to the small deviant, as compared with the ADHD group.

#### Conventional ANOVA Analyses for MMN, P3a, and LDN

##### Lexical tone

**Figure 5:**
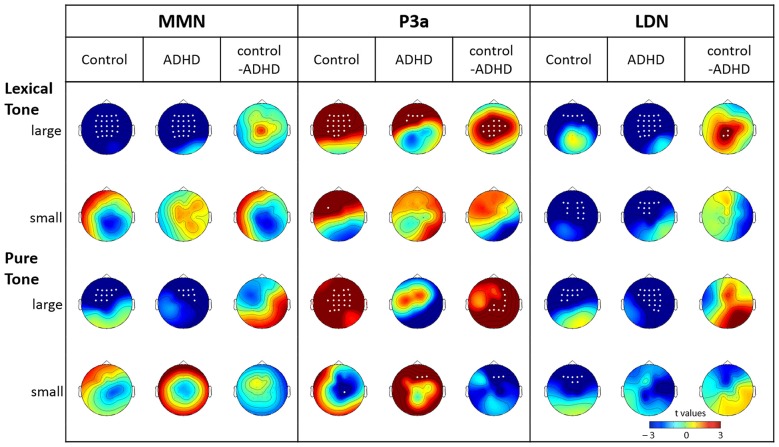
**Topographic maps for the significance of mismatch negativity (MMN), P3a, and late discriminative negativity (LDN) (*t*-value) elicited by large and small deviant lexical tones and pure tones and the planned group comparison for each of them**. White dots indicate electrodes with a significant difference between the standard and deviant or a significant group effect (*p* < 0.05).

The analysis of MMN time windows revealed a significant main effect of condition (*F*_(2,58)_ = 23.07, *p* < 0.001, η^2^ = 0.85) and interaction between condition and electrode (*F*_(48,1392)_ = 3.76, *p* < 0.01, η^2^ = 0.11). The planned comparison, as shown in Figure 5, indicated that, both groups showed significant MMN to the large deviant, but no significant mismatch response to the small deviant, for almost every electrode. No group effect was found for either large or small lexical tone changes in the MMN time window.

The analysis of the P3a time window also revealed a significant main effect of condition (*F*_(2,58)_ = 3.96, *p* < 0.05, η^2^ = 0.12) and a significant interaction between condition and electrode (*F*_(48,1392)_ = 4.85, *p* < 0.001, η^2^ = 0.14). For the large lexical tone deviant, significant P3as were found at almost every electrode in the control group, but were restricted to five electrodes in frontal sites in the ADHD group. The significant group effect indicated that the control group showed more positive P3a than the ADHD group did at 12 frontal-to-posterior electrodes. As for the small lexical tone deviant, the control group showed significant P3a at FC3, while the ADHD group did not reveal any P3a. No group effect was found for the small lexical tone deviant.

The analysis of the LDN time window revealed a significant main effect of condition (*F*_(2,58)_ = 10.11, *p* < 0.001, η^2^ = 0.26) and a significant interaction between condition and electrode (*F*_(48,1392)_ = 2.75, *p* < 0.05, η^2^ = 0.09). The interaction between group and electrode was marginally significant (*F*_(48,1392)_ = 2.42, *p* = 0.06, η^2^ = 0.08). The planned comparison showed that, for the large deviant, significant LDN in the control group was restricted to frontal sites. However, the ADHD group showed significant LDN at almost every electrode. The group effect indicated that the ADHD group showed more negative LDN than the control group did in central-to-posterior sites. As for the small deviant, both groups showed significant frontal-to-central LDN. No group effect was found at any electrode.

##### Pure tone

The analysis of MMN time window revealed a significant main effect of condition (*F*_(2,58)_ = 9.16, *p* < 0.001, η^2^ = 0.24) and a marginally significant main effect of group (*F*_(2,58)_ = 3.99, *p* = 0.055, η^2^ = 0.12). There were also a significant condition-electrode interaction (*F*_(48,1392)_ = 29.79, *p* < 0.001, η^2^ = 0.16) and a marginally significant group-electrode interaction (*F*_(48,1392)_ = 2.66, *p* = 0.07, η^2^ = 0.08). The planned comparisons revealed significant MMN to the large deviant in both control and ADHD groups. Moreover, the control group exhibited a more left-lateralized frontal-to-central MMN, while the ADHD group showed a more right-lateralized frontal-to-central MMN. Although the group effect was not significant, the topographic map showed that the control group tended to elicit more negative MMN in left frontal-to-central sites than the ADHD group did. As for the small deviant, neither did both groups yield any significant MMN, nor was there any significant group effect.

In the P3a time window, there was a marginally significant condition-group interaction (*F*_(48,1392)_ = 2.91, *p* = 0.06, η^2^ = 0.09). The planned comparison revealed that, when responding to the large deviant, P3a was only found in the control group, and not in the ADHD group. The group comparison indicated that the control group elicited more positive P3a than the ADHD group did, mainly in right central-to-posterior sites. For the small deviant, the control group did not show any positivity, yet the ADHD group showed significant positivity at Fz, F2, and F4. The group comparison confirmed that the ADHD group elicited more positive amplitude than the control group did at these three electrodes.

Regarding the LDN analysis, there was a significant main effect of condition (*F*_(2,58)_ = 20.15, *p* < 0.001, η^2^ = 0.41), a significant condition-electrode interaction (*F*_(48,1392)_ = 3.64, *p* < 0.01, η^2^ = 0.11), and a significant group-electrode interaction (*F*_(48,1392)_ = 4.54, *p* < 0.01, η^2^ = 0.13). The three-way interaction among group, electrode and condition was marginally significant (*F*_(48,1392)_ = 1.88, *p* = 0.09, η^2^ = 0.06). Similar to the findings for lexical tone, for the large deviant of pure tone, the significant LDN in the control group was restricted to frontal-to-central sites. However, the ADHD group showed LDN at almost every electrode (except for Pz, P1, P3, CP3 and C3). Although the group comparison did not reveal a significant difference, the topographic group effect map suggested that the ADHD group tended to elicit more negative LDN than the control group did, especially in the right central-to-posterior sites. For the small deviant, the control group elicited significant LDN in frontal sites, while no significant LDN was noted in ADHD. However, no significant group effect was found at any electrode.

#### Correlational Analysis

Based on the results of cluster-based analysis and conventional ANOVA analysis, the most reliable group effect was that the control group showed a larger P3a in response to large lexical tone changes than the ADHD group did. However, our participants covered a wide range of ages from 6 to 15 years old. Thus, we performed further correlational analysis to examine whether the mean amplitudes of P3a for each participant in each channel correlated with participants’ characteristics, including age and the scores for ADHD-related symptoms—eight scores from the DSM-5 criteria and SNAP-IV Rating Scale for ADHD-related symptoms (see Table 1). Only those that yielded significant correlation coefficients in at least three adjacent channels would be considered to be significant predictors.

**Figure 6:**
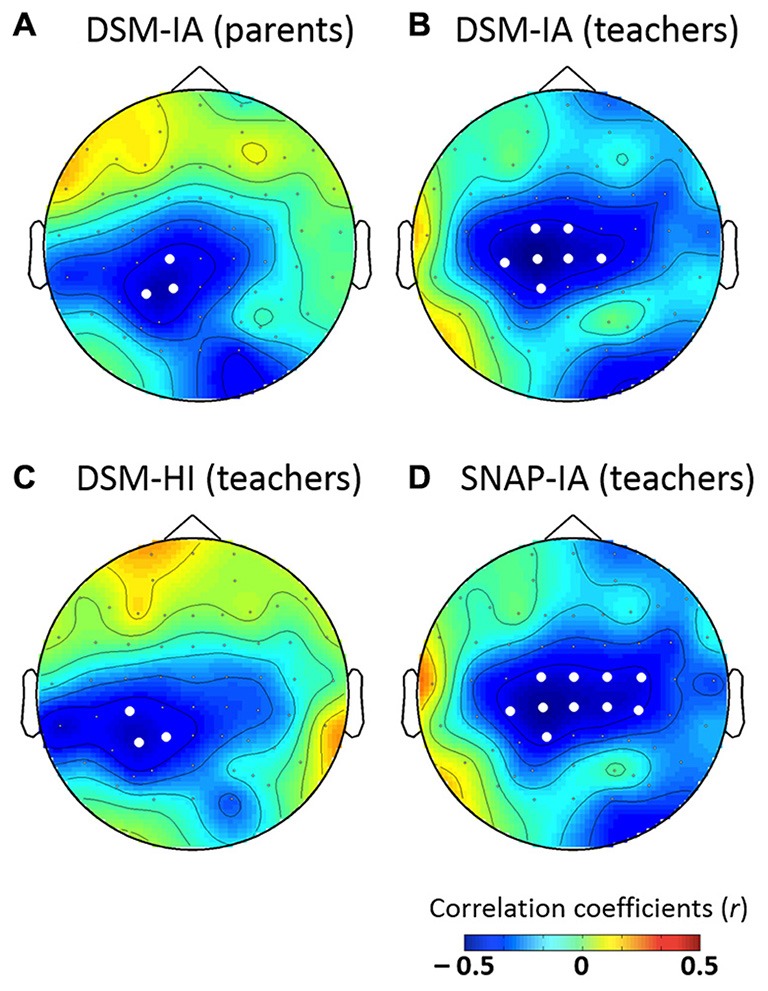
**Topographic maps for the significance of correlation between P3a with (A) DSM-IA (parents), (B) DSM-IA (teachers), (C) DSM-HI (teachers), and (D) SNAP-IA (teachers)**. White dots indicate electrodes with significant correlations (*p*s < 0.05).

The results revealed no significant correlation between age and P3a. However, the mean amplitudes of P3a were negatively correlated with the DSM-IA scores of parents as well as three scores of teachers: SNAP-IA, DSM-IA and DSM-HI. The topographic maps of the Pearson’ correlation coefficients are shown in Figure 6, in which electrodes showing significant correlations (*p*s < 0.05) are represented with white dots. In summary, P3a was negatively correlated with DSM-IA scores (patents; *r*_max_ = −0.437 at P1, *p* < 0.05), DSM-IA scores (teachers; *r*_max_ = −0.482 at CP1, *p* < 0.01), DSM-HI scores (teachers; *r*_max_ = −0.434 at P3, *p* < 0.05), and SNAP—IA scores (teachers; *r*_max_ = −0.474 at CP1, *p* < 0.01) in clusters with central distribution. Children with higher scores on those ADHD-related symptoms tended to show less positive P3a.

## Discussion

This study examined auditory change detection ability for lexical tones and pure tones, as indexed by MMN, P3a, and LDN components, in children with ADHD and their age-matched controls. Across the cluster-based analysis and the conventional ANOVA analysis, we found no difference in MMN between children with and without ADHD. The most reliable group difference was that the ADHD group showed a reduced P3a for the large deviant of lexical tone. Meanwhile, the conventional analysis also identified a reduced P3a and an enhanced LDN for the large deviant of pure tone in the ADHD group. In addition, P3a activities were negatively correlated with a set of behavioral measures, including the DSM-IA scores of parents, and teacher scores on SNAP-IA, DSM-IA, and DSM-HI. To summarize, children with higher ADHD tendency showed less positivity on P3a. The role of these ERP components in identifying attentional deficits in ADHD will be further discussed below.

For lexical tone changes, both the ADHD and control groups revealed typical MMN, P3a, and LDN for the large deviant (T1/T3). As for the small deviant (T2/T3), the control group showed a positive deflection at 200–250 ms (based on the cluster-based analysis) and at 300–400 ms (based on the conventional time-window analysis) and LDN, while the ADHD group only elicited significant LDN. The overall pattern of the control group is in line with that seen in the study by Lee et al. (2012), which used the same set of stimuli in preschoolers and showed that the large deviant elicited an MMN followed by a P3a between 300 and 400 ms, while the small deviant elicited a P-MMR between 200 and 350 ms with no preceding MMN. It is unclear whether the significant positivity identified in the control group in this study is a P-MMR, like that found by Lee et al. (2012), which would reflect more difficult discrimination, or a P3a, reflecting an involuntary switch of attention to novelty. In fact, Lee et al. (2012) suggested that there might be two types of positivity: P-MMR and P3a. The elicitation of the latter requires a sufficient difference between deviant and standard, and its amplitude increases with the size of the deviance (Escera et al., 1998; Jakoby et al., 2011). Jakoby et al. (2011) demonstrated that the latency of MMN correlated significantly with that of P3a, and proposed that earlier pre-attentive discrimination ability, indexed by MMN, might result in an earlier attention switch, indexed by P3a, to the deviant stimuli. Therefore, in the current findings, the positivity following MMN for the large lexical tone change was considered to indicate P3a. Furthermore, the reduced P3a for the large deviant lexical tone and the pure tone in ADHD group suggested a deficit in it in the context of involuntary attentional switch.

The other possibility is that the late positivity for the small deviant in the control group might represent a delayed P-MMR, as P-MMR has been associated with difficult discrimination (Lee et al., 2012). The positive and negative responses can be seen simultaneously in infants and preschoolers and the latency of the adult-like MMNs shifts earlier with increasing age, which might lead them to overlap partially or fully, eliminating each other (Morr et al., 2002; Maurer et al., 2003). P-MMR decreases in amplitude with increasing age, and is generally absent by 8 years of age (Datta et al., 2010; Shafer et al., 2010), although it can still be found in older children or adults with very fine-discrimination (Ahmmed et al., 2008; Kuo et al., 2014). In general, the adult-like MMN/P3a response pattern becomes more prominent, whereas the slow positive difference wave diminishes as age increases (He et al., 2007). This implies that, at a specific point in maturation, P-MMR and MMN might offset each other and result in an isoelectric response for a specific contrast. The absence of mismatch responses to the small deviant contrast might result because the participants in this study were older (age ranged between 9 and 15 years old) than the ones in the studies by Lee et al. (2012). However, we were not sure whether the late positivity between 300 and 400 ms for the small deviant should be considered as a P3a (without preceding MMN) or a delayed P-MMR. Further studies are needed to clarify this question.

The data from pure tone changes showed a similar pattern. For the large deviant, both groups elicited significant MMN and LDN. Again, the two groups showed no difference on MMN, while the ADHD group tended to elicit more enhanced LDN than the control group did (based on significance in cluster-based analysis in the time window of 500–550 ms; a similar trend could also be found in the conventional analysis). Most importantly, the P3a was only found in the control group, not in the ADHD group. The group effect was found in the conventional analysis, which showed that the ADHD group elicited a smaller P3a than the control group did. As for the small deviant, neither MMN nor P3a, but only LDN, was found in both groups.

A major characteristic of MMN theory is that the underlying process and the associated neural response are automatic, meaning that the elicitation of MMN can be considered attention-independent (Näätänen et al., 2007). Across findings from lexical tone and pure tone experiments, our data confirmed that the passive auditory oddball paradigm elicited MMN, P3a, and LDN (or RON), implying that deviant stimuli were processed with different involvement of attention at different stages. Studies have suggested that these three components differ in the nature of attentional control applied. The MMN could be elicited pre-attentively, and its elicitation could be considered attention-independent (Näätänen et al., 2007); on the other hand, P3a, an index for the switch of attention, and LDN, an index for subsequent reorienting back to stimuli for further evaluation, are attention-dependent. For example, Berti and Schröger (2003) manipulated the working memory load using the passive auditory oddball paradigm, and found that MMN was not modulated by task load; on this basis, they suggested that working memory did not exert top-down control over the pre-attentive change detection system at the initial stage. However, P3a and RON both declined with increased task load. The present study revealed that neither group showed any difference in their MMN time windows, whether for lexical tone or pure tone. However, the ADHD group showed attenuated P3a and enlarged LDN in detecting large pure tone and lexical tone changes. This suggests that attentional deficits in ADHD are not specific to linguistic or non-linguistic stimuli, and that this is reflected in involuntary attention switch, indexed by P3a, and in attention reorienting for further evaluation, indexed by LDN/RON.

However, previous studies on ADHD have shown controversial and conflicting findings on P3a. Some studies found enhanced P3a in response to a novel sound in ADHD groups (Kilpeläinen et al., 1999a; Gumenyuk et al., 2005). However, Law et al. (2013) found smaller P3a responses to Cantonese tonal stimulus in native speakers of Cantonese with poor perception of tones, and suggested instead that P3a may indicate lower cognitive capability in attention switching, auditory attention, or memory. Kemner et al. (1996) reported smaller P3 amplitude at the parietal midline in response to auditory deviance in children with ADHD. P3 amplitude that was significantly larger with methylphenidate than with a placebo has also been reported (Winsberg et al., 1997). This reduced P3a to large deviants of lexical tones and pure tones in this study suggests impaired involuntary attention switching in children with ADHD. ERP data from an auditory oddball task have been shown to differentiate children with AHDH from healthy controls with 73.3% accuracy, with parietal-occipital P3 amplitude to the target and standard stimuli contributing most to this classification (Smith et al., 2003). This is further supported by our correlational analysis, in which children with higher ADHD tendency, indexed by parents’ and teachers’ rating on ADHD symptoms, tended to show less positive P3a amplitude when responding to large deviant lexical tone.

The present study also observed an enhanced LDN for ADHD, especially for large deviant lexical tone. The functional signature of LDN has been less addressed in the literature, especially for ADHD. Gumenyuk et al. (2005) found that LDN had smaller amplitude and shorter latency in children with ADHD. Elsewhere in the literature, the reduced amplitude of LDN has been associated with prefrontal cortical dysfunction in attentional orientation, and the decreased latency of LDN, with a higher degree of impulsivity. For example, Kilpeläinen et al. (1999b) reported attenuated LDN elicited by pure-tone deviants and suggested deficits in the frontal-mediated mechanism for auditory sensory memory as an explanation. A study among children with a family risk of dyslexia showed enhanced LDN to the frequency change, correlated particularly with verbal short-term memory (Hämäläinen et al., 2015). LDN amplitude was adopted to reflect further discriminative processing of the deviant stimulus, in addition to the automatic change detection reflected by MMN at the pre-attentive stage (Hämäläinen et al., 2008). The present finding of enhanced LDN amplitude over the frontal scalp in the ADHD group, as compared to that in controls, might suggest that children with ADHD engage in more extensive stimulus evaluation, and take greater effort in evaluation and formation of long-term representations of detected stimuli, because of their abnormal distractibility.

## Conclusion

To our knowledge, this is the first study applying both lexical and pure tones in auditory ERP tasks in order to investigate auditory perception and processing in children with ADHD. By using the multiple-deviants oddball paradigm, this study examined how individuals detected and reacted to unexpected auditory changes in the sensory environment at different stages of attentional control, including pre-attentive change detection, indexed by MMN; involuntary orienting of attention, indexed by P3a; and attention reorienting for further evaluation reflected by LDN. The ADHD group showed attenuated P3a and enhanced LDN for large deviants, across both pure tone and lexical tone changes, when compared with age-matched controls. However, the two groups did not differ in MMN, whether for lexical tone or pure tone. These findings suggest that children with ADHD have deficits in both involuntary orienting and attention reorienting while processing auditory deviation. However, children with ADHD have no problem with pre-attentive processing. Thus, in dealing with the academic problems of children with ADHD, this impairment of attentional control to auditory change detection should be considered. Moreover, these ERP components may serve as neural markers to evaluate attentional control and basic auditory discrimination in children with developmental disorders, and to evaluate the effectiveness of training programs or medications (such as mehthylphenidate and atomoxetine) to alleviate these dysfunctions.

## Conflict of Interest Statement

The authors declare that the research was conducted in the absence of any commercial or financial relationships that could be construed as a potential conflict of interest.
